# Uptake of ^18^F-FET and ^18^F-FCH in Human Glioblastoma T98G Cell Line after Irradiation with Photons or Carbon Ions

**DOI:** 10.1155/2017/6491674

**Published:** 2017-01-16

**Authors:** Francesca Pasi, Marco Giovanni Persico, Federica Eleonora Buroni, Carlo Aprile, Marina Hodolic, Franco Corbella, Rosanna Nano, Angelica Facoetti, Lorenzo Lodola

**Affiliations:** ^1^Department of Oncohaematology, Radiotherapy Unit, Fondazione IRCCS Policlinico San Matteo, Viale Golgi 19, 27100 Pavia, Italy; ^2^Department of Biology and Biotechnology “Lazzaro Spallanzani”, University of Pavia, Via Ferrata 9, 27100 Pavia, Italy; ^3^Department of Oncohaematology, Nuclear Medicine Unit, Fondazione IRCCS Policlinico San Matteo, Viale Golgi 19, 27100 Pavia, Italy; ^4^Scuola Universitaria Superiore IUSS Pavia, Pavia, Italy; ^5^National Centre for Oncological Hadrontherapy (CNAO), Via Campeggi 53, 27100 Pavia, Italy; ^6^Nuclear Medicine Research Department, IASON GmbH, Graz, Austria

## Abstract

The differential diagnosis between recurrence of gliomas or brain metastases and this phenomenon is important in order to choose the best therapy and predict the prognosis but is still a big problem for physicians. The new emerging MRI, CT, and PET diagnostic modalities still lack sufficient accuracy. Radiolabeled choline and amino acids have been reported to show great tumor specificity. We studied the uptake kinetics of [^18^F]fluoromethyl-choline (FCH) and O-(2-[^18^F]fluoroethyl)-L-tyrosine (FET) by the T98G human glioblastoma cells from 20 to 120 min after irradiation either with photons at 2-10-20 Gy or with carbon ions at 2 Gy (at the National Centre for Oncological Hadrontherapy (CNAO), Pavia, Italy). We also evaluated the cell death and morphology changes induced by radiation treatment. Both FET and FCH are able to trace tumor behavior in terms of higher uptake for increased doses of radiation treatment, due to the upregulation of cells attempts to repair nonlethal damage. Our data suggest that both FCH and FET could be useful to analyze the metabolic pathways of glioblastoma cells before and after radiotherapy. Physicians will have to consider the different kinetics pathways of uptake concerning the two radiopharmaceuticals.

## 1. Introduction

The differential diagnosis between recurrence of gliomas and brain metastases and the phenomenon of radiation necrosis plays an important role in both therapeutic and prognostic settings. The distinction between tumor recurrence and radionecrosis is important in order to choose the best subsequent therapy and prognosis is guided by the cause of progression. Necrosis is an important histological feature of glial tumors. Tumor necrosis is ischemic in nature, due to an insufficient blood supply [1]. The differential diagnosis is complicated by the fact that late radionecrosis appears at various times after treatment, from 6 months up to several years [2]. Moreover, radionecrosis phenomenon is observable without the direct involvement of the brain in the field of radiation (bystander effect, e.g., head and neck cancer irradiated with hadrons). Nowadays, despite the enormous improvement of diagnostic modalities, including the various applications of MRI, CT, and PET, the diagnosis and grading of primary brain tumours lack sufficient accuracy. This is more relevant when recurrence after therapy, early neuroinflammation [3], or late radionecrosis is concerned [4]. Despite the technological advances and new MR investigable parameters [5], there is a wide area that does not allow the reasonable accuracy that the clinicians need. Also, PET radiopharmaceuticals (^11^C-METH, ^18^F-FET, ^18^F-FCH, and ^18^F-DOPA) have limitations mainly in terms of specificity, although the dynamic investigation, only possible with the fluorinated compounds (^18^F *t*_1/2_ = 108 min), appears to offer good results [6, 7]. The discrimination between tumor and inflammation is not possible with ^18^F-FDG because it is not a specific tumor marker. Radiolabeled choline and amino acids have been reported to show greater tumor specificity than ^18^F-FDG, both in experimental animal models and in humans [8]. Other PET tracers which are considered as proliferation markers may allow an improved differential diagnosis between tumor and inflammation [9, 10].

The aim of our research was to evaluate the uptake of two different radiopharmaceuticals ([^18^F]fluoromethyl-choline (FCH) and O-(2-[^18^F]fluoroethyl)-L-tyrosine (FET)) by glioblastoma cells in basal conditions and after irradiation with photons (conventional LINAC) and hadrons (carbon ions).

**Figure 1:**
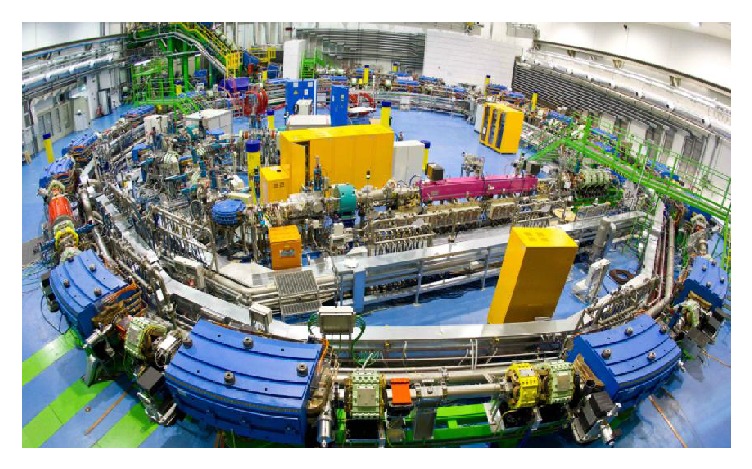
View of the CNAO synchrotron and beam transport lines.

We studied the uptake kinetics by the T98G human glioblastoma cells from 20 to 120 min after incubation with FCH and FET. Then, we carried out the evaluation of the uptake changes of the T98G cell line after irradiation either with photons at 2-10-20 Gy (at the Radiotherapy Unit, Fondazione IRCCS Policlinico San Matteo, Pavia, Italy) or with carbon ions at 2 Gy (at the National Centre for Oncological Hadrontherapy (CNAO), Pavia, Italy) (Figure 1).

## 2. Materials and Methods

### 2.1. Cell Culture

Human glioblastoma T98G cells were obtained from the European Collection of Cell Cultures (Porton Down, Salisbury, UK). T98G cells were cultured in Eagle's minimum essential medium (EMEM; Euroclone SpA, MI, Italy) supplemented with 10% foetal bovine serum (Sigma-Aldrich, St. Louis, MO, USA), 100 units/mL of penicillin/streptomycin (Euroclone SpA, MI, Italy), 2 mM L-glutamine (Euroclone SpA, MI, Italy), and 0.01% sodium pyruvate (Sigma-Aldrich, St. Louis, MO, USA) at 37°C in an atmosphere of 5% CO_2_. Stock cultures were maintained in exponential growth as monolayers in 75 cm^2^ Corning plastic tissue-culture flasks (VWR International PBI Srl, MI, Italy).

### 2.2. Irradiation Treatments

**Figure 2:**
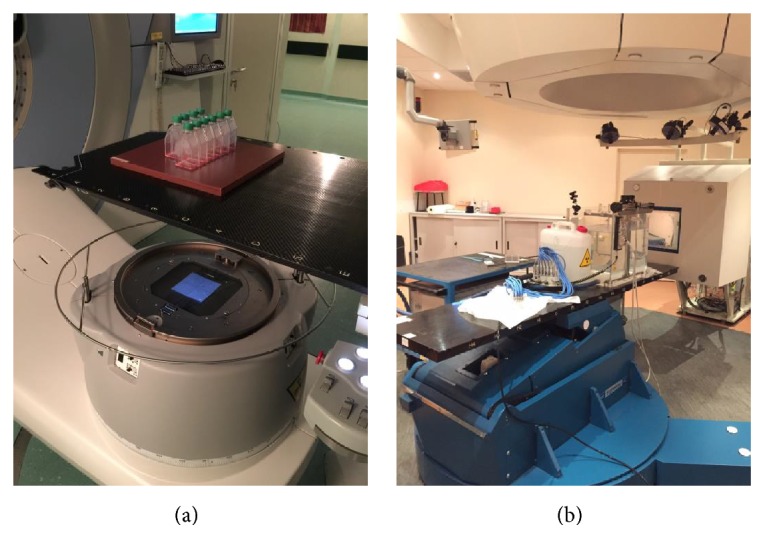
Experimental setup used for T98G cells irradiation at the Radiotherapy Unit (a) and at CNAO (b).

Cells were irradiated at doses of 2 Gy, 10 Gy, and 20 Gy with photons at room temperature using a LINAC at 6 MeV (ELEKTA Synergy; Radiotherapy Unit, Fondazione IRCCS Policlinico San Matteo, Pavia, Italy) with a dose rate of 3 Gy/min. The flasks containing the cells were placed vertically at the isocenter and a 5 mm thick plastic sheet was placed below the flask surface to allow dose buildup (Figure 2(b)). Cells irradiation with carbon ions (energy range: 246–312 MeV/u) was performed using the synchrotron clinical beam at the National Centre for Oncological Hadrontherapy (CNAO) in Pavia, Italy [11]. The flasks were placed vertically in a water phantom on the patient's couch, so that the surface at which the cells were stuck was at 15 cm depth, corresponding to the mid Spread-Out Bragg Peak (SOBP), where the SOBP was obtained with active beam modulation. Carbon ions Linear Energy Transfer (LET) in the mid SOBP, evaluated with a Monte Carlo Fluka simulation, was 40 keV/*μ*m. The irradiation dose was 2 Gy (physical dose) for each sample (dose rate of 0.7 Gy/min) (Figure 2(a)) [12].

Sham-irradiated cells (0 Gy) were considered as control.

An hour before irradiation, the medium was removed from the flasks and a fresh medium was added to the cells. Cells were replaced in an incubator at 37°C after irradiation treatment.

### 2.3. Radioactive Tracer Incubation

^18^F-FCH and ^18^F-FET were obtained from IASON GmbH (Graz-Seiersberg, Austria). Cells, seeded at a density to obtain 2 × 10^5^ cells per flask when irradiation treatments were performed, grew adherent to the plastic surface at 37°C in 5% CO_2_ in complete medium. Irradiation treatment was performed 20 h after the cell seeding. Radioactive tracer experiments were performed 36 hours after irradiation. The medium was renewed before performing the studies. Cells were incubated at 37°C with 100 kBq (100 *μ*L) equimolar amounts of ^18^F-FCH or ^18^F-FET, added to 2 mL of medium in each flask for varying incubation times (20, 40, 60, 90, and 120 min or 20, 40, 60, 80, 100, and 120 min according to availability of cells) under 5% CO_2_ gaseous conditions. Radiotracer incubation was done in complete medium. Control samples underwent the same procedure as other samples, but they were incubated with 100 *μ*L of saline instead of a radiotracer.

### 2.4. Cell Kinetic Studies and Uptake Evaluation

The cellular radiotracer uptake was determined with a 3 × 3′′ NaI (Tl) pinhole 16 × 40 mm gamma counter (Raytest, Straubenhardt, Germany). All measurements were carried out under the same counting position along with a standardized source to verify the counter's performance and the data were corrected for background and decay. Total radioactivity was counted when the radiotracer was added to the medium in each flask (time 0). After 20, 40, 60, 90, and 120 min or 20, 40, 60, 80, 100, and 120 min from time 0, the medium was harvested, the cells were rapidly washed three times with 1 mL of phosphate-buffered saline (PBS), and radiopharmaceutical uptake for each sample was assessed. The uptake measurements are expressed as the percentage of the administered dose of tracer per 2 · 10^5^ cells after correction for negative control uptake (flasks containing no cells with complete medium and incubated with a radiopharmaceutical).

### 2.5. Cell Viability Assay

At the end of quantitative gamma spectrometry, adherent cells were harvested with 1% trypsin-EDTA solution and supernatants with adherent cells were counted with Burker's chamber. Trypan Blue dye assay was performed to assess cell viability as standard protocol. Finally, we calculated the % of cell viability: number of cells adherent to the flask after irradiation versus the nonirradiated samples.

### 2.6. Cell Death Analysis and Morphology Evaluation

To evaluate the cell death and morphology changes induced by radiation treatment, May–Grunwald–Giemsa staining [13] and Annexin V-propidium iodide (Ann-PI) [14] assays were performed on irradiated T98G cells. 2 · 10^4^ cells were seeded on coverslip slides 24 hours before treatments. Cells were irradiated at 2, 10, and 20 Gy and after 36 hours from treatment the slides were stained. A group of T98G cells were sham-irradiated and handled in parallel with irradiated cells.

T98G cells were stained with May–Grunwald–Giemsa stain (Sigma-Aldrich, St. Louis, MO, USA) according to standard protocol and examined under 400x power using a light microscope (Leica DM LB2; Wetzlar, Hesse, Germany). This qualitative analysis evaluates the morphology, the shape, and the density of cells. The Ann-PI test, carried out according to the manufacturer's instructions (eBioscience, San Diego, CA, USA), is based on fluorescence technique that allows distinguishing viable, damaged, or dead cells. In radiobiology, they can be used to identify cell damage induced by radiation. The membrane status can be monitored using some specific probes like Annexin V and PI. Permeability depends on the physicochemical characteristics of the fluorochrome molecule and on the function of the cell membrane that can indicate the viability of cells. This analytical approach discriminates live cells from damaged ones, through correlated analysis of the two fluorescent signals acquired with parallel observations with phase contrast microscopy. In particular, the dual staining with Annexin V and PI permits identifying the different levels of damage in a cell population that could include viable cells with intact membrane (no or little fluorescence), early damaged cells with partial disruption of membrane (green fluorescence), and late damaged cells with a loss of membrane integrity that could lead to cell death (both red and green fluorescence). These populations were distinguished easily on an Olympus BX51 microscope with standard fluorescence equipment.

The evaluation of the mean number of the Annexin/PI double labelled cells was obtained by counting cells of about ten microscopic fields. We reported the percentages of damaged cells in each irradiated and nonirradiated (control) sample.

With this approach, samples of cells can be analyzed in a short time with minimal cell manipulation, avoiding the use of trypsin that could produce additional damage and could affect the results. Moreover, May–Grunwald–Giemsa staining was used on the same samples as a rapid and routinary method to evaluate cell morphology and the presence of damaged cells.

In vitro experiments were conducted in duplicate and repeated twice. All values are expressed as mean values with confidence interval (CI 95%). The uptake of radiotracer is represented as a function of the incubation period; all values are shown in figures as a percentage of the administered dose per 2 · 10^5^ cells (mean ± CI 95%). Therefore, if error bars on the *y*-axis do not overlap, the two points are considered significantly different.

## 3. Results

### 3.1. Radiopharmaceuticals Binding Assay

**Figure 3:**
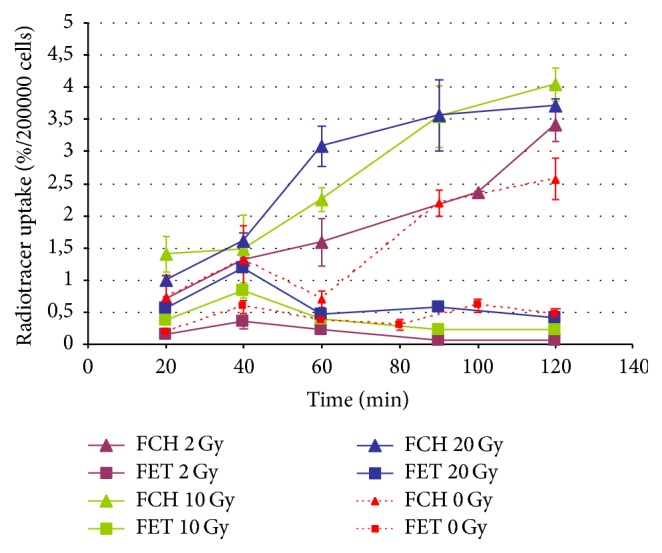
Uptake of FCH and FET by T98G cells after irradiation with photons at different doses compared to controls (0 Gy).

Figure 3 shows the comparison between FCH uptake and FET uptake by T98G cells after photons irradiation (2-10-20 Gy); 0 Gy curves (controls) are reported as red dotted lines. FCH uptake increased linearly with the incubation time, and the absolute values of radiotracer uptake (%/2 · 10^5^ cells) were enhanced with the raising of the radiation dose. The same trend was found in the kinetic curves regarding FET uptake, but the shape of the curves remained unchanged in irradiated compared to nonirradiated samples reported in Figure 3. The uptake of FET is lower than of FCH, especially after 60 min since the radiopharmaceutical incubation.

**Figure 4:**
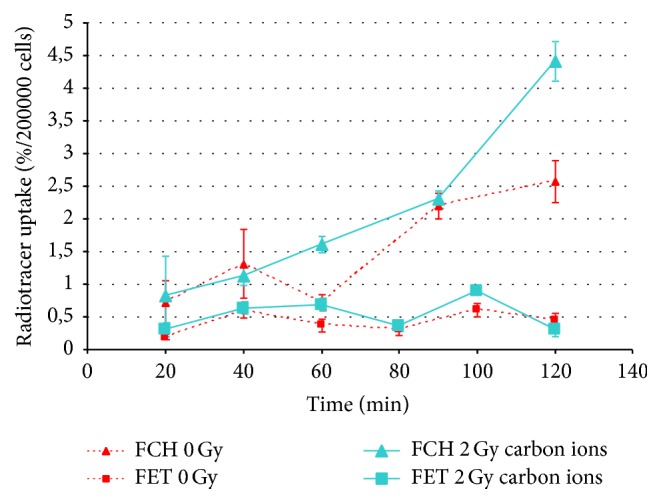
Uptake of FCH and FET by T98G cells after irradiation with carbon ions compared to control (0 Gy).

Figure 4 shows the comparison of the kinetic curves of FCH and FET uptake in T98G cells. Red lines represent the uptake relative to nonirradiated samples. The light blue lines represent the uptake of irradiated (2 Gy of carbon ions) samples.

For the “0 Gy” curves (red dotted lines), the highest uptake of FCH was observable at 90 and 120 min. The percentage uptake of FET in comparison to FCH was lower by a factor of more than 3, but the two kinetic curves were different. In fact, while FCH curves showed a progressive rise reaching a maximum after 120 min, FET showed more rapid initial uptake up to 40 min.

After the irradiation with carbon ions, T98G cells constantly increased the uptake of FCH along the incubation time with a final peak after 120 min, while the kinetic curve relative to FET uptake was quite similar to the nonirradiated samples curve, even if the radiotracer uptake (%/2 · 10^5^ cells) was a bit higher at 60 and 100 min incubation time.

**Figure 5:**
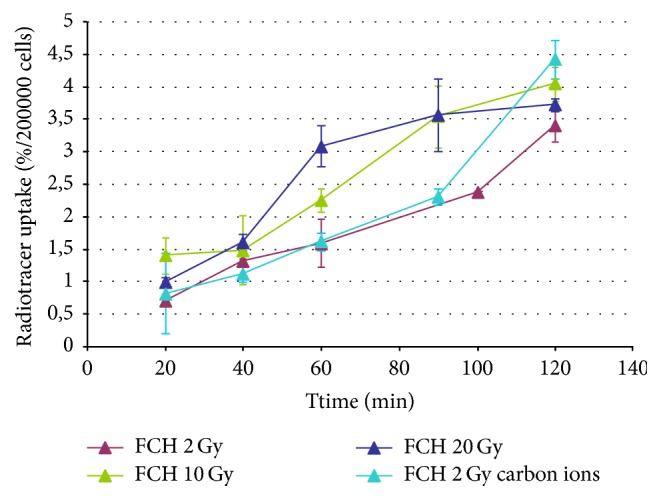
Uptake of FCH by T98G cells after irradiation with photons and carbon ions.

Figure 5 illustrates the comparison of FCH uptake in T98G cells after photons or carbon ions irradiation. Irradiation of the cells with 2 Gy carbon ions seemed to lead to a similar result of 2 Gy photons treatment, except for 120 min incubation time where the maximum uptake of FCH by T98G was reached with carbon ions.

**Figure 6:**
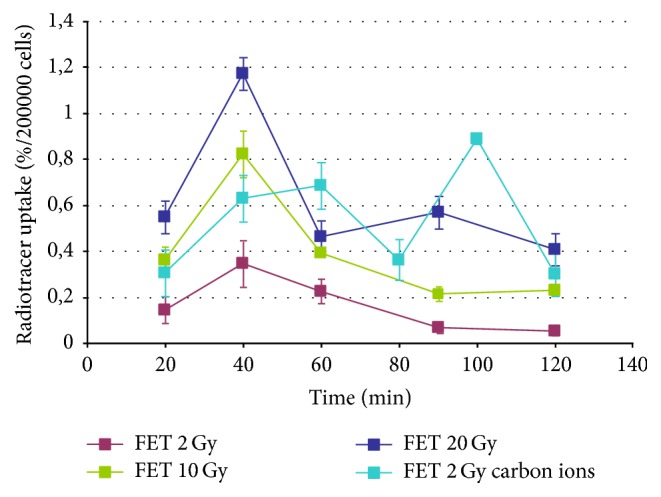
Uptake of FET by T98G cells after irradiation with photons and carbon ions.

Figure 6 shows the uptake of FET by T98G cells after irradiation with photons or carbon ions (2-10-20 Gy or 2 Gy, resp.). The curves showed less evident uptake of FET after 2 and 10 Gy delivered by photons than after 2 Gy delivered by carbon ions. The kinetics curves were also different, with a peak at 100 min in the 2 Gy carbon ions irradiated samples instead of the 40 min peak observable in the photons irradiated ones.

As a negative control, flasks containing medium without cells, incubated under similar conditions, showed a nonsignificant presence of radiotracers.

### 3.2. Cell Viability

**Figure 7:**
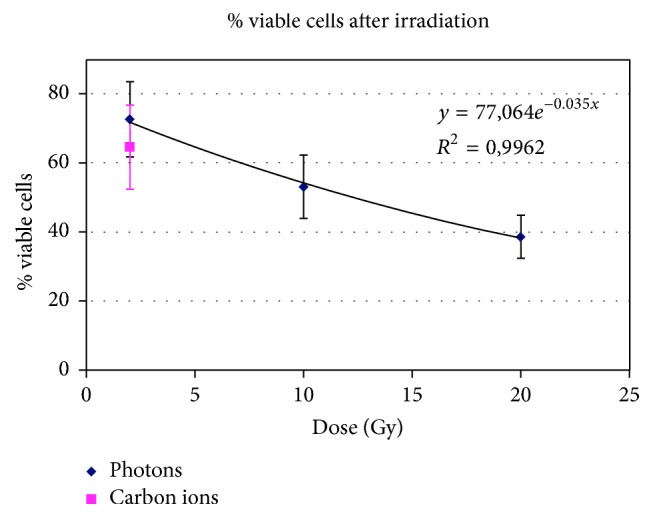
Cell viability after irradiation with photons (2-10-20 Gy) and carbon ions (2 Gy).

The values of cell viability (cells adherent to the flasks versus nonirradiated samples) after irradiation with photons were 73% ± 11% (2 Gy), 53% ± 9% (10 Gy), and 39% ± 6% (20 Gy), while after irradiation with 2 Gy of carbon ions the value was 65% ± 12% (Figure 7). Exposure to the gaseous mixture was maintained throughout the experiment and the cell viability (assessed by Trypan Blue assay for each flask) was calculated to be approximately 90% under all experimental conditions (data not shown).

### 3.3. Cell Death Analysis and Morphology Evaluation

These techniques were applied to samples to assess the damage of different doses of radiation.

**Figure 8:**
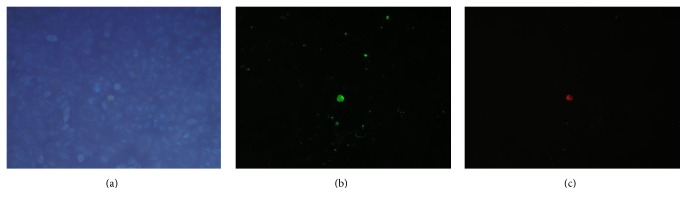
Annexin-PI treatment on control T98G cells: (a) whole control cells, (b) Annexin V positive cells, and (c) propidium iodide positive cells (magnification 100x).

Control cells (0 Gy) were Annexin V and PI negative, confirming that cells were viable with intact membrane. In fact, as shown in Figures 8(a), 8(b), and 8(c), only one cell per microscopy field of view was detectable as damaged with Annexin-PI technique.

**Figure 9:**
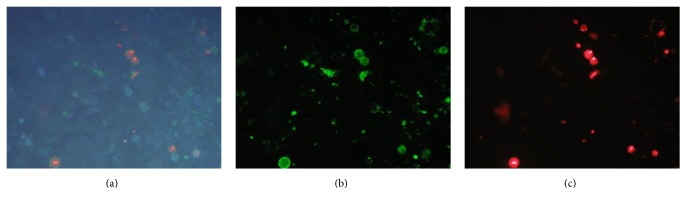
Annexin-PI treatment on 20 Gy irradiated T98G cells: (a) whole irradiated cells, (b) Annexin V positive cells, and (c) propidium iodide positive cells (magnification 100x).

An increased number of fluorescent cells were noticed in cells treated with 20 Gy photon radiation, suggesting that this dose induced more consistent damage: an increase in the amount of green and red fluorescent cells, respectively, Annexin V and PI positive, was observed compared with control (Figures 9(a), 9(b), and 9(c)). Taking account of the values of cell viability after irradiation, the percentage of damaged cells was 21% after 20 Gy treatment, 9% after 10 Gy, and 4% after 2 Gy compared with the 3% of the 0 Gy control cells (CI_95%_  ±3%).

**Figure 10:**
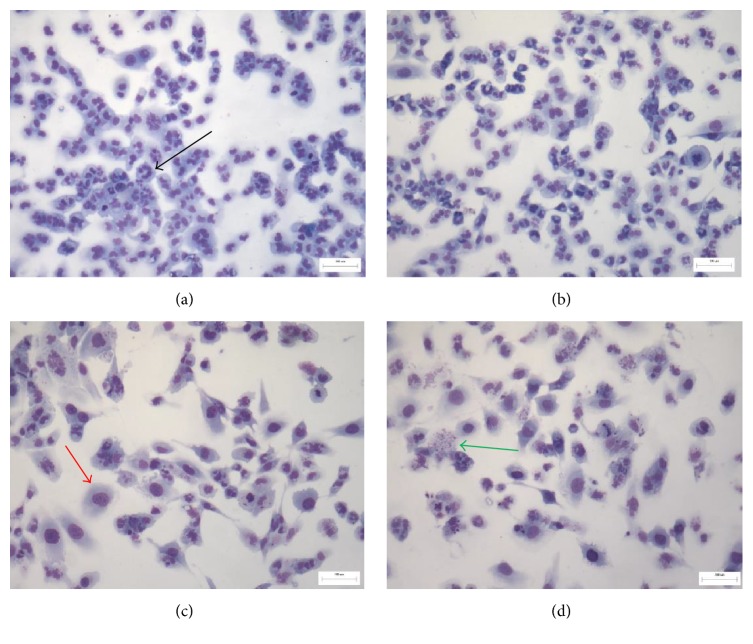
May–Grunwald–Giemsa staining of irradiated T98G cells at different doses: (a) control cells (0 Gy), (b) 2 Gy, (c) 10 Gy, and (d) 20 Gy (magnification 100x). Scale bar reported in figures: 100 *μ*m. Black arrow: an example of polynucleated cell. Red arrow: an example of giant cell. Green arrow: an example of damaged cell with karyorrhexis.

May–Grunwald–Giemsa stain (Figure 10(a)) shows that control cells were polynucleated in active proliferation with cell heterogeneity, in the presence of different cell morphology, which are typical characteristics of T98G cells. Irradiated samples show a decrease of cell number with the increase of dose radiation, suggesting that radiation causes detachment of dead cells from the surface (Figures 10(b), 10(c), and 10(d)).

Moreover, in irradiated cells, the presence of damaged cells with karyorrhexis and karyolysis was observed as well as an increase in the number of giant cells as a consequence of damage induced by radiation treatments (Figures 10(b), 10(c), and 10(d)). They appeared to increase dependently on dose radiation and seemed to be not reversible and repairable at 20 Gy (Figure 10(d)).

## 4. Discussion

The aim of our research project is to investigate in vitro the different radiopharmaceuticals uptake by T98G glioblastoma cells, involved in the development of radiation necrosis, after different irradiation conditions with photons or carbon ions.

The in vitro model used in our experiments allows the direct comparison of different radiopharmaceuticals as potential candidates for neurooncological PET imaging. In our previous study [15], we compared the uptake of FCH and FDG by T98G glioblastoma cells and fibroblasts. The results showed superiority of FCH in terms of absolute uptake and an optimal target to nontarget ratio in the brain, whereas the major limitation of FDG is its physiological parenchymal uptake. The study proved the efficacy of FCH, better than FDG in establishing the tumor-to-background ratio in brain tumors. However, direct translation to clinical application is hampered by certain conflicting results reported in the literature [9, 16].

Subsequently, we tested the FET affinity for neoplastic tissue, confirming its potential as a viable oncological PET marker [17].

FET could be more useful in the presence of reparative changes after therapy, where the higher affinity of FCH to inflammatory cells makes it more difficult to distinguish between tumor persistence and nonneoplastic, radiochemotherapy related changes [18].

In this work, we evaluated the uptake of FCH and FET with different metabolic aspects, in basal conditions and after irradiation with photons (conventional LINAC) or hadrons (carbon ions).

The goal of our experiment was to evaluate the uptake of two radiotracers used in the diagnosis and follow-up of glioblastoma after radiotherapy, in other words to evaluate in which way the radiation delivered to tumor cells can modify the uptake and metabolism of FCH and FET. This aspect has a deep impact on PET imaging, where early and late radionecrosis phenomena can constitute a diagnostic dilemma [19], frequently unresolved by the other diagnostic modalities as MRI and CT.

The radiation dose we used, either of photons or of carbon ions, largely exceeded the limit of increased sensitivity to acute dose described as low-dose hyperradiosensitivity (HRS) and is in the range of increased radioresistance (IRR) where the radiation damage leads to the activation of a repair process and follows a linear model [20]. In fact, data relative to the number of cells adherent to the flask 36 hours after irradiation and 2 hours after incubation with the radiopharmaceutical fit exactly (*R*^2^ = 0.996) with an exponential equation (77.06*e*^−0.035*D*^) (Figure 7).

The FCH uptake after photons irradiation (Figure 4) tends to increase as dose increases, and the uptake pattern is more linear with time, still increasing at the end of observation (2 hours) without the transient washout at 60 minutes we previously described [15, 17] in nonirradiated cells. Such uptake pattern is about the same if we consider the 2 Gy curve of both photons and carbon ions (Figures 5 and 6); however, the uptake at the end of observation is about 1% higher with carbon ions.

The FET uptake after photons (Figure 5) irradiation shows a marked decrease at 2 Gy, while after 10 Gy irradiation it is about the same for nonirradiated cells and increases markedly after 20 Gy irradiation. The effect after 2 Gy irradiation of carbon ions is less evident, characterized by small, albeit significant uptake increasing without change of the time activity curve.

A direct comparison of the effect of the two different radiation sources is hampered by the fact that only 2 Gy data are available for carbon ions. However, the more pronounced effect of carbon ions could be related to higher LET [20].

Despite the fact that the number of viable cells, adherent to the surface of the flask, decreases with the increasing of radiation dose, along with major evidence of altered membrane permeability and morphological changes, the uptake of both tracers we studied tends to increase. From these results, it appears that the cells, which survived from the treatment, seem to take more radiopharmaceuticals for high doses of radiation compared to low doses and control. This phenomenon can be due to the fact that these radioresistant cells are highly reactive and able to capture more of FCH for the membrane metabolism.

Methods based on cell membrane permeability using fluorescent probes are the most established and are routinely used in many applications [14]. As our results suggest, radiation injury could induce increased permeability of membrane. Cells may respond to radiation injury with increased activities which may be regarded as stimulation secondary to a preceding injury. Some of the responses of this kind are involved in the processes of recovery from injury and can result in visible changes in cells [21, 22].

It is possible to postulate that viable (adherent) cells enhance their mechanisms of repair and resistance. Millet and coworkers [23] showed in the same cell line that radiation upregulates telomerase activity, demonstrating a PIK3/AKT-independent pathway of telomerase activation. In the literature, radiation seems to be responsible for the upregulation of choline kinase and/or choline transporter [24, 25] as well as of L-amino acid transporter LAT 1 [26].

It is interesting to note that even if the absolute uptake per flask is taken into account (data not shown) without normalization to 2 · 10^5^ cells, the same uptake pattern is recognizable, characterized by increased uptake of both radiopharmaceuticals, further confirming the upregulation mechanism.

These upregulation mechanisms can affect the interpretation of PET images obtained with both radiopharmaceuticals in the assessment of response to radiotherapy, indicating either a tumor burden larger than the one actually present or, more likely, the persistence of a subpopulation of cancer cells with acquired radioresistance when a dosage is in the range of increased radioresistance (IRR).

Our data suggest that FCH and FET could be useful to analyze different metabolic pathways of glioblastoma cells before and after radiotherapy in accordance with Galldiks et al. [27]. The paradox effect on FET uptake after irradiation has been previously described in MCF7 cell line [28]. Many factors could be involved including p53mt expression and selection of more aggressive radioresistant clones.

Further studies shall be conducted to study the uptake of radiopharmaceuticals by irradiated cells after a longer time between irradiation and analysis to assess over time the phenomenon of radiation-induced damage repair.

## 5. Conclusions

The differential diagnosis between tumor recurrence and early and late radionecrosis constitutes a real dilemma for physicians. Both FET and FCH are able to trace tumor behavior in terms of higher uptake for increased doses of radiation treatment, attributable to the upregulation of cells attempts to repair nonlethal damage. This phenomenon appeared to be more evident for FCH due to the role of choline in increased membrane metabolism. Our data suggest that both FCH and FET could be useful to analyze the metabolic pathways of glioblastoma cells before and after radiotherapy, providing information on their greater or lower metabolic status.

In order to indirectly translate our results into clinical application, physicians will have to consider the different kinetics pathways of uptake concerning the two radiopharmaceuticals. The future step will be to test the behavior of other cell types such as neoplastic, endothelial, and microglia cells incubated with medium harvested from these irradiated cells, containing inflammatory and growth factors, cytokines, receptor ligands, and other factors that could contribute to the development of radiation necrosis. The new experiments will let us study the effect of these inflammation media on distant radionecrosis-neuroinflammation development.
